# *Moringa oleifera* Alkaloids Inhibited PC3 Cells Growth and Migration Through the COX-2 Mediated Wnt/β-Catenin Signaling Pathway

**DOI:** 10.3389/fphar.2020.523962

**Published:** 2020-11-12

**Authors:** Jing Xie, Feng-xian Luo, Chong-ying Shi, Wei-wei Jiang, Ying-yan Qian, Ming-rong Yang, Shuang Song, Tian-yi Dai, Lei Peng, Xiao-yu Gao, Liang Tao, Yang Tian, Jun Sheng

**Affiliations:** ^1^ College of Food Science and Technology, Yunnan Agricultural University, Kunming, China; ^2^ Engineering Research Center of Development and Utilization of Food and Drug Homologous Resources, Ministry of Education, Yunnan Agricultural University, Kunming, China; ^3^ National Research and Development Professional Center for Moringa Processing Technology, Yunnan Agricultural University, Kunming, China; ^4^ College of Science, Yunnan Agricultural University, Kunming, China; ^5^ Yunnan Province Engineering Research Center of Functional Food of Homologous of Drug and Food, Yunnan Agricultural University, Kunming, China; ^6^ Key Laboratory of Pu-er Tea Science, Ministry of Education, Yunnan Agricultural University, Kunming, China

**Keywords:** *Moringa oleifera* alkaloids, prostate cancer, PC3 cells, cell growth and migration, COX-2-wnt/β-catenin signaling pathway

## Abstract

*Moringa oleifera* Lam. (*M. oleifera*) is valuable plant distributed in many tropical and subtropical countries. It has a number of medicinal uses and is highly nutritious. *M. oleifera* has been shown to inhibit tumor cell growth, but this effect has not been demonstrated on prostate cancer cells. In this study, we evaluated the inhibitory effect of *M. oleifera* alkaloids (MOA) on proliferation and migration of PC3 human prostate cancer cells *in vitro* and *in vivo*. Furthermore, we elucidated the mechanism of these effects. The results showed that MOA inhibited proliferation of PC3 cells and induced apoptosis and cell cycle arrest. Furthermore, MOA suppressed PC3 cell migration and inhibited the expression of matrix metalloproteinases (MMP)-9. In addition, MOA significantly downregulated the expression of cyclooxygenase 2 (COX-2), β-catenin, phosphorylated glycogen synthase 3β, and vascular endothelial growth factor, and suppressed production of prostaglandin E_2_ (PGE_2_). Furthermore, FH535 (β-catenin inhibitor) and MOA reversed PGE_2_-induced PC3 cell proliferation and migration, and the effects of MOA and FH535 were not additive. *In vivo* experiments showed that MOA (150 mg/kg) significantly inhibited growth of xenograft tumors in mice, and significantly reduced the protein expression levels of COX-2 and β-catenin in tumor tissues. These results indicate that MOA inhibits the proliferation and migration, and induces apoptosis and cell cycle arrest of PC3 cells. Additionally, MOA inhibits the proliferation and migration of PC3 cells through suppression of the COX-2 mediated Wnt/β-catenin signaling pathway.

## Introduction

Prostate cancer is the second most common malignant tumor in men worldwide, and the fifth leading cause of cancer-related death (Siegel et al., 2017). In more than 180 countries around the world, the incidence of prostate cancer has increased from 1.10 million to 12.76 million from 2012 to 2018 (Torre et al., 2015; Ferlay et al., 2019). Development of diagnostic techniques has improved the screening rate of prostate cancer, but clinical treatment strategies are limited by slow progress of basic science research. Traditional treatments such as prostate cancer hormone blocking therapy and surgery can significantly improve the survival of hormone-dependent patients. However, no effective treatments exist for hormone-independent prostate cancer.

Cyclooxygenase (COX) is a key rate-limiting enzyme involved in conversion of arachidonic acid to prostaglandins (PG). There are three COX subtypes, including COX-1, COX-2, and COX-3. COX two plays an important role in tumor cell growth, invasion, and metastasis through regulation of PGE_2_ synthesis (Singh and Katiyar, 2013). Moreover, PGE_2_ can activate the GSK3β/β-catenin pathway via G-protein coupled receptors (EP2 and EP4), resulting in transcription of oncogenes such as c-myc, cyclin D1, and vascular endothelial growth factor (VEGF), and growth and migration of tumor cells. In addition, a number of studies have reported that COX-2 was highly expressed in prostate cancer and stimulated prostate cancer cell proliferation (Gupta et al., 2000; Dandekar and Lokeshwar, 2004; Richardsen et al., 2010). Therefore, regulation of the expression of COX-2 and its downstream signaling pathways has received increased attention as a target for treatment of prostate cancer.

Development of novel anti-tumor drugs from natural sources has received increased interest in recent years. *Moringa oleifera* Lam. (*M. oleifera*) is one of the Moringaceae, and it is a valuable plant distributed in many tropical and subtropical countries (Abdull Razis et al., 2014). A large number of studies have shown that *M. oleifera* induces strong anti-proliferative effects, and induces apoptosis in human hepatoma cells (Sadek et al., 2017), cervical cancer cells (Jafarain et al., 2014), human oral epidermoid carcinoma cells (Sreelatha et al., 2011), breast cancer cells, and colon cancer cells (Al-Asmari et al., 2015). Alkaloids are a class of organic compounds with nitrogen-containing moieties that have been shown to exert antitumor effects. Studies have shown that methanolic extracts of *M. oleifera* inhibited proliferation of U266B1 human multiple myeloma cells, A549 lung cancer cells, HepG2 liver cancer cells, HT-29 colon cancer cells, and IM-32 human neuroblastoma cells, and alkaloids are believed to exert these effects (Elsayed et al., 2015). However, the molecular mechanisms of *M. oleifera* alkaloid (MOA)-induced inhibition of growth and migration of prostate cancer cells have not been characterized. The present study investigated the role of MOA in inhibition of growth and migration of PC3 prostate cancer, and explored the potential mechanisms underlying these effects.

## Materials and Methods

### Preparation of *M. oleifera* alkaloids

The leaves of *M. oleifera* was obtained from Yunnan Tianyou Technology Development Co., Ltd. in Dehong Prefecture, Yunnan Province, China (Batch No. 20190001S), and identified by Professor Jiang-miao Hu (Kunming Institute of botany, Chinese Academy of Sciences). A voucher specimen (No. YSTY-14) was deposited in the Engineering Research Center of development and utilization of Food and Drug Homologous Resources, Ministry of Education, Yunnan Agricultural University, Kunming, China. *M. oleifera* leaf powder (10 kg) was extracted three times with 50% ethanol for 24 h each time. The extracts were filtered, combined, concentrated, and the ethanol was evaporated. The aqueous solution obtained following concentration was adjusted to pH 2 with 10% HCl, then extracted three times with ethyl acetate. The acidified water solution was adjusted to pH 10 using a sodium hydroxide solution and extracted three times with chloroform. The chloroform extracts were combined, and the chloroform was evaporated to yield 30 g of alkaloids (0.3% yield, w/w).

### Cell Lines and Culture

Ten cancer cell lines (U251, A431, A375, Hela, PC3, HepG2, MDA-MB-231, HuTu80, HCT116, and HT29) and human normal prostate epithelial RWPE-1 cells were purchased from the Chinese Academy of Science (Shanghai, China). The cells were cultured in DMEM High Glucose, 1:1 DMEM:F12 or RPMI 1640 medium (HyClone, Novato, CA, United States) supplemented with 10% fetal bovine serum (BI, CA, United States) and penicillin-streptomycin (Solarbio, Beijing, China) in a 5% CO_2_ incubator maintained at 37°C.

### Cell Viability Assay

Ten cancer cell lines (U251, A431, A375, Hela, PC3, HepG2, MDA-MB-231, HuTu80, HCT116, and HT29; 1 × 10^4^ cells/well) were cultured in 96-well plates. When cells reached 90% confluence, they were treated with MOA (0, 20, 40, 80, 160, or 320 μg/ml) for 48 h, and untreated cells served as controls (0 μg/ml). Twenty microliters of MTT (Sigma-Aldrich, Saint Louis, MO, United States) solution was added to each well to a final concentration of 5 mg/ml, and the cells were incubated for 4 h. Following incubation, the media was discarded. Two hundred microliters of DMSO was added to each well, and the plates were shaken for 10 min. Absorbance was measured at 492 nm. The half maximal inhibitory concentration (IC_50_) values were calculated as the drug concentrations necessary to inhibit 50% proliferation as compared to untreated control cells.

The PC3 cells and human normal prostate epithelial RWPE-1 cells (1 × 10^4^ cells/well) were seeded in 96-well plates for 24 h and treated with different concentrations of MOA (0, 20, 40, 80, 160 or 320 μg/ml) for 24, 48, or 72 h. After 24, 48, and 72 h, the cell viability was evaluated by the MTT assay according to the manual.

The PC3 cells (1 × 10^4^ cells/well) were seeded in 96-well plates for 24 h. PC3 cells were pretreated with PGE_2_ (20 μM) for 1 h, then with β-catenin inhibitor FH535 (2.5 μM) for 1 h, and finally treated with MOA (40 μg/ml) for 48 h. After 48 h, the cell viability was evaluated by the MTT assay according to the manual.

### Colony Formation Assay

PC3 cells and human normal prostate epithelial RWPE-1 cells (1,000 cells/well) were cultured in 6-well plates (NEST, Jiangsu, China), then treated with MOA (0, 20, 40, or 80 μg/ml) for 24, 48, or 72 h. After culturing for 15 days, the cells were fixed in methanol and stained with 0.1% crystal violet for 15–30 min. After washing multiple times, the plates were allowed to dry, the cells were photographed, and crystal (Aladdin, Shanghai, China) violet was dissolved using 10% glacial acetic acid (Aladdin, Shanghai, China). Absorbance was measured at 560 nm, and statistical analysis was performed.

### Analysis of Apoptosis and Cell Cycle Using Flow Cytometry

The PC3 cells and human normal prostate epithelial RWPE-1 cells were seeded in 6-well plates at a density of 2 × 10^5^ cells/well, then treated with MOA (0, 20, 40, or 80 μg/ml) for 48 h. The cells were harvested, washed twice with PBS, and centrifuged. Then, 100 μl of Annexin-V/FITC and PI dye (Sigma, United States) was added, and the cells were incubated for 20 min at room temperature in the dark. The cells were analyzed using flow cytometry (BD, FACSCalibur, CA, United States). The percentage of apoptotic cells was determined using FlowJo software (Treestar, United States).

The PC3 cells were seeded in 6-well plates at a density of 2 × 10^5^ cells/well, then treated with MOA (0, 20, 40, or 80 μg/ml) for 48 h. The cells were fixed in 70% ethanol overnight at 4°C in a refrigerator, then incubated with RNase-containing PI dye (Yeasen, Shanghai, China) for 30 min at 37°C. Cell cycle was evaluated using flow cytometry (BD, FACSCalibur, United States). The percentage of cells in each cell cycle stage was determined using FlowJo software (Treestar, United States).

### Wound Healing Migration Assay

PC3 cells were seeded in 60 mm plates at a density of 1 × 10^6^ cells/plate. The cells were allowed to adhere, and a sterilized micro-pipette tip was used to scratch across the cell layer. The cells were then treated with MOA (0, 20, or 40 μg/ml) for 48 h. The scratches were photographed using an inverted microscope at the beginning and end of the experiment, and the results were analyzed using Image-pro and GraphPad Prism 5. Each experiment was repeated independently in triplicate.

### Cell Migration Assay

Two hundred microliters of PC3 cells suspensions (serum-free medium containing 3 × 10^5^ cells) containing different concentrations of MOA (0, 20, or 40 μg/ml) were added to the upper chamber of a transwell apparatus. Eight hundred microliters media containing 10% FBS with different concentrations of MOA was added to the lower chambers. The cells were incubated for 48 h, and the cells were washed with PBS three times, fixed in methanol for 30 min, stained with 0.1% crystal violet (Aladdin, Shanghai, China) for 30 min, then visualized and photographed. The cells were then incubated in 33% glacial acetic acid for 10 min, and absorbance was measured at 570 nm.

Two hundred microliters of PC3 cells suspensions (serum-free medium containing 3 × 10^5^ cells) containing PGE_2_ (20 μM), β-catenin inhibitor FH535 (2.5 μM), or different concentrations of MOA (0, 20, or 40 μg/ml) were added to the upper chamber of a transwell apparatus. Eight hundred microliters media containing 10% FBS with different concentrations of MOA was added to the lower chambers. The cells were incubated for 48 h, the cell migration rate was evaluated by the cell migration assay according to the manual.

### Western Blot Analysis

PC3 cells were collected after 48 h of treatment with different concentrations of MOA (0, 20, or 40 μg/ml), and total protein was extracted using RIPA buffer. Protein concentration was measured using a BCA protein assay kit (Beyotime, Shanghai, China). Proteins were separated on 10% SDS polyacrylamide gels and transferred to 0.45 μm polyvinylidene fluoride membranes (Millipore, MA, United States). The membranes were blocked with 5% skim milk (BD, United States) for 60 min, then incubated with primary antibodies against Bax, Bcl-2, CyclinE, p21, MMP9 (1:500, Wanglei biology, Shenyang, China), COX-2, β-catenin (1:1,000, abcam, MA, United States), GSK-3β, phosphorylated glycogen synthase 3β (P-GSK-3β), VEGF (1:200, Santa Cruz Biotechnology, CA, United States), and β-actin (1:1,000, abcam, MA, United States) overnight at 4°C. The membranes were washed with PBST three times, then incubated for 1 h with HRP-conjugated secondary IgG antibody (1:10,000, R&D Systems, United States), and the signal was detected using ECL western blotting substrate (Proteinsimple, FluorChem E).

Tumor tissues were collected and total protein was extracted using RIPA buffer, the protein levels of COX-2 and β-catenin were determined by Western blotting.

### Confocal Fluorescence Microscopy

Human PC3 cells (2 × 10^6^) were seeded in confocal dishes (Corning, United States), and incubated with MOA (0, 20, or 40 μg/ml) for 48 h. The cells were fixed in 4% paraformaldehyde for 20 min at room temperature, washed three times with PBS, and permeabilized with PBS containing 0.5% Triton-X-100 for 15 min at room temperature. The cells were then washed three times with PBS. Five percent BSA was added to the cells, and they were incubated overnight at 4°C. The cells were then incubated with a β-catenin antibody (1:50, Abcam, San Francisco, CA, United States) overnight at 4°C, washed three times, then incubated with a secondary antibody (goat Alexa 647 and mouse IgG, 1: 200, Abcam, CA, United States) for 1 h at room temperature. Then, DAPI was added and the cells were incubated at room temperature for 15 min to stain the cell nucleus. Cells were visualized using a confocal fluorescence microscope (Olympus Corporation, FV500).

### Prostaglandin E_2_ Immunoassay

PC3 cells (1 × 10^6^/plates) were seeded in 60-mm plates and treated with MOA (0, 20, or 40 μg/ml) for 48 h. The cells were collected, and total protein was extracted using RIPA lysis buffer and quantified. The levels of PGE_2_ in the cell lysates were measured using enzyme linked immunosorbent assay (ELISA) (MEIMIAN, Jiangsu, China) according to the manufacturer’s instructions.

### Xenograft Model in Nude Mice

Eighteen male BALB/C nude mice (5–6 weeks old) were purchased from Changzhou Cavans Laboratory Animal Co., Ltd. and all mice were subcutaneously injected with PC3 cells (4 × 10^6^ cells per mouse). After 2 weeks, the mice were randomly divided into three groups of six mice each, including a control group (saline), an MOA-150 mg/kg group, and an MOA-300 mg/kg group. All mice underwent oral gavage for 26 days, and tumor size was measured every 2 days using a caliper (volume mm^3^ = length × width × width/2). The mice were then euthanized with CO_2_, and tumor tissues were excised, and stored in formalin at −80°C until use. The animal experiment protocols were reviewed and approved by the Institutional Animal Ethical Committee of Yunnan Agricultural University (IACUC-20190410-26).

### Immunohistochemistry Assay

Tumor tissues were embedded in paraffin and sectioned (3 μm). The sections were dehydrated and subjected to antigen retrieval using 0.01 M sodium citrate. The sections were then placed in 3% H_2_O_2_ solution and incubated for 25 min. After blocking for 30 min with 5% BSA, the sections were incubated with primary antibodies [anti-COX2 and β-catenin (1:100, Wang lei biology, Shenyang, China)] overnight. On the next day, the sections were incubated with horseradish peroxidase-linked secondary antibodies for 30 min, stained with 3,3′-diaminobenzidine (DAB) for 2 min, then counterstained with hematoxylin for 1 min. Photographs were taken using an Olympus microscope (Olympus, 4M05688).

### Statistical Analysis

All data were presented as the mean ± standard error of the mean (SEM) of at least three replicate experiments. Statistical analysis was performed using GraphPad Prism 5 (San Diego, CA, United States). Differences between two groups were analyzed using Student’s t test and differences among three or more groups were analyzed using one-way ANOVA. *p* < 0.05 was considered statistically significant.

## Results

### *M. oleifera* alkaloids Inhibited Proliferation of PC3 Cells

**FIGURE 1:**
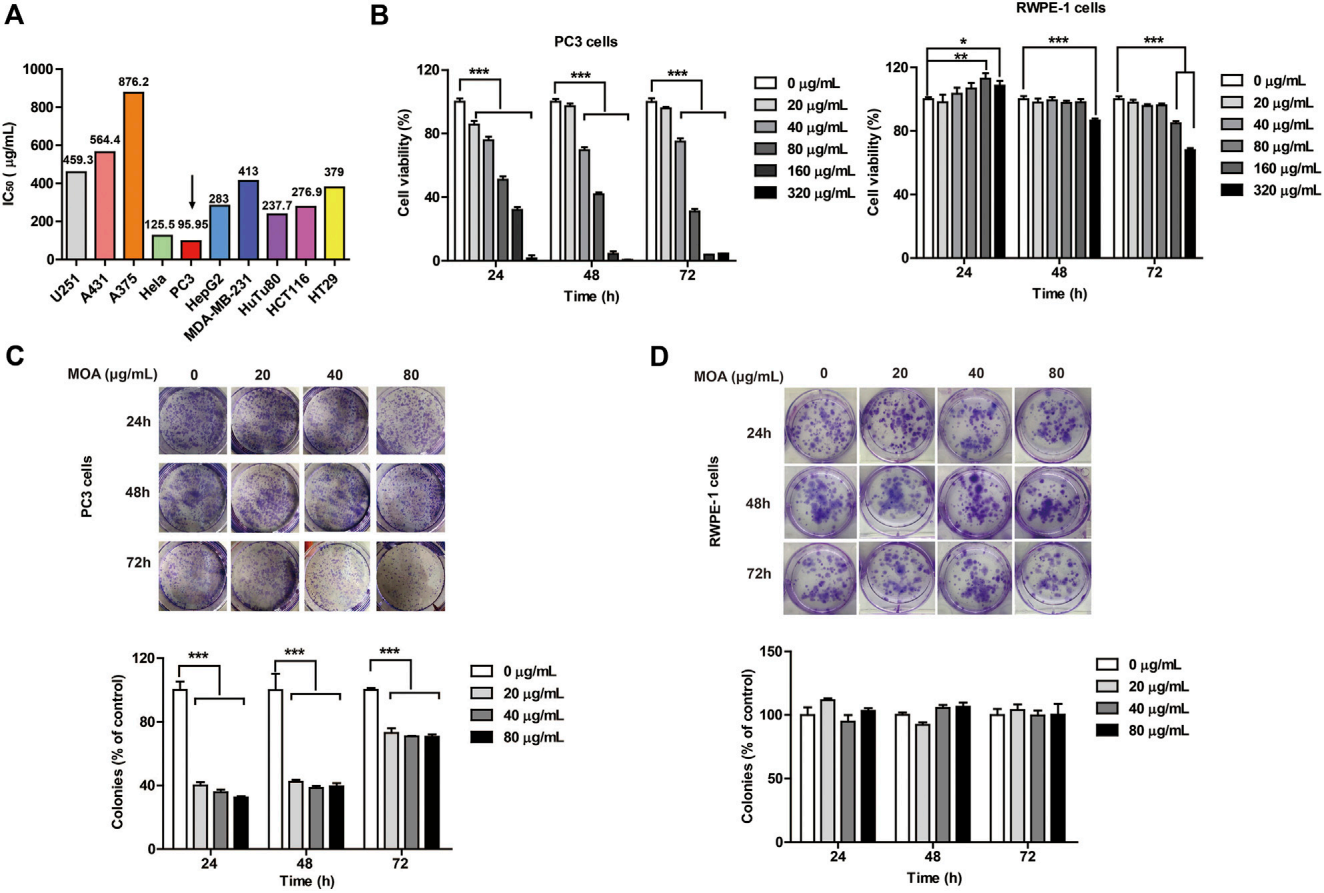
The effect of MOA on the proliferation of PC3 and RWPE-1 cells. **(A)** The half maximal inhibitory concentration (IC_50_) of ten tumor cell lines following treatment with MOA (0–320 μg/ml) for 48 h. **(B)** Cell viability of PC3 and RWPE-1 cells following MOA (0–320 μg/ml) treatment for 24, 48, and 72 h **(C,D)** Analysis of the colony formation ability of PC3 and RWPE-1 cells following treatment with MOA (0, 20, 40, and 80 μg/ml) for 24, 48, and 72 h. The cellular colony formation rates are expressed as fold changes. Results are expressed as the mean ± SEM of three independent experiments. *p < 0.05, **p < 0.01, ***p < 0.001 vs. 0 μg/ml.

To determine the anti-cancer effects of MOA, 10 cancer cell lines (U251, A431, A375, Hela, PC3, HepG2, MDA-MB-231, HuTu80, HCT116, and HT29) were treated with MOA (0, 25, 50, 100, 200, 400, or 800 μg/ml) for 48 h. IC_50_ of the cell lines were 459.3, 564.4, 876.2, 125.5, 95.95, 283, 413, 237.7, 276.9, and 379 μg/ml, respectively (Figure 1A). These results demonstrated that MOA induced a selective inhibitory effect against PC3 cells.

To further investigate the effects of MOA on human prostate cancer PC3 cells and human normal prostate epithelial RWPE-1 cells proliferation, PC3 and RWPE-1 cells were treated with MOA (0, 20, 40, 80, 160, or 320 μg/ml) for 24, 48, and 72 h, then analyzed using the MTT and colony formation assays. The results showed that MOA decreased viability of PC3 cells in a dose-dependent manner (Figure 1B). Colony formation assay results indicated that MOA significantly decreased the number of colonies of PC3 cells compared to the control group (*p* < 0.001) (Figure 1C). However, when the dose of MOA was as high as 80 μg/ml, it had no effect on the cell viability and cloning rate of RWPE-1 cells (Figures 1B,D). These results demonstrated that MOA significantly reduced proliferation of PC3 cells, but has no effect on normal cells.

### *M. oleifera* alkaloids Induced PC3 Cell Apoptosis and Cell Cycle Arrest

**FIGURE 2:**
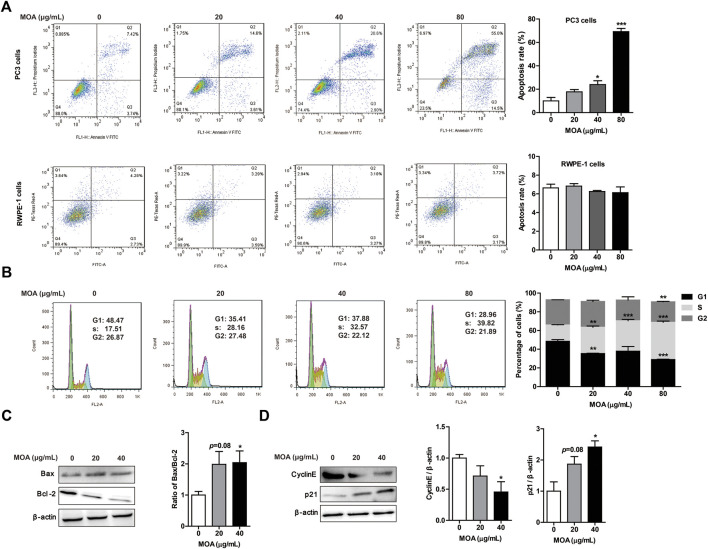
Treatment with MOA induced PC3 cell apoptosis and cell cycle arrest. PC3 and RWPE-1 cells were treated with MOA (0, 20, 40, and 80 μg/ml) for 48 h. The apoptosis of PC3 and RWPE-1 cells **(A)**, and cell cycle of PC3 cells **(B)** were determined by flow cytometry. The apoptosis proteins Bax and Bcl-2 **(C)**, and the cell cycle proteins cyclin E and p21 **(D)** of PC3 cells were detected by western blotting, with β-actin as a loading control. Quantification of relative Bax, Bcl-2, cyclin E, and p21 protein levels. Results are expressed as the mean ± SEM of three independent experiments. *p < 0.05, **p < 0.01, and ***p < 0.001 vs. 0 μg/ml.

To determine whether MOA inhibited growth of PC3 cells due to increased apoptosis and cell cycle arrest, we analyzed the cell cycle profile and determined the percentage of apoptotic cells following MOA treatment. The results showed that MOA increased the apoptosis rate of PC3 cells in a dose-dependent manner. Specifically, the percentage of apoptotic cells following treatment with 40 μg/ml and 80 μg/ml for 48 h resulted in 23.95 ± 3.25% (*p* < 0.05) and 69.24 ± 2.76% (*p* < 0.001) apoptotic cells, respectively, which was significantly greater than that in control cells (10.00 ± 2.98%). However, MOA has no effect on the apoptosis rate of RWPE-1 cells (Figure 2A). Furthermore, the percentage of S phase cells in PC3 cells increased from 17.15 ± 0.19% in control cells to 28.16 ± 1.24% (*p* < 0.01), 32.57 ± 1.36% (*p* < 0.001), and 39.82 ± 1.23% (*p* < 0.001) in response to treatment with 20, 40, and 80 μg/ml MOA for 48 h, and the percentage of cells in G1 phase decreased from 48.47 ± 1.76% in control cells to 35.41 ± 0.33% (*p* < 0.01), 37.88 ± 5.03%, and 28.96 ± 0.37% (*p* < 0.001) in response to treatment with 20, 40, and 80 μg/ml MOA for 48 h (Figure 2B).

We also evaluated the expression levels of proteins associated with apoptosis and the cell cycle in PC3 cells using western blotting. As shown in Figure 2C, MOA increased the expression of Bax and decreased the expression of Bcl-2, which caused a significantly increased ratio of Bax to Bcl-2. Furthermore, MOA significantly increased the expression of p21 and decreased the expression of CyclinE in a dose-dependent manner (Figures 2C,D). These results suggested that MOA inhibited cell proliferation by inducing apoptosis and cell cycle arrest in PC3 cells.

### *M. oleifera* alkaloids Inhibited PC3 Cell Migration

**FIGURE 3:**
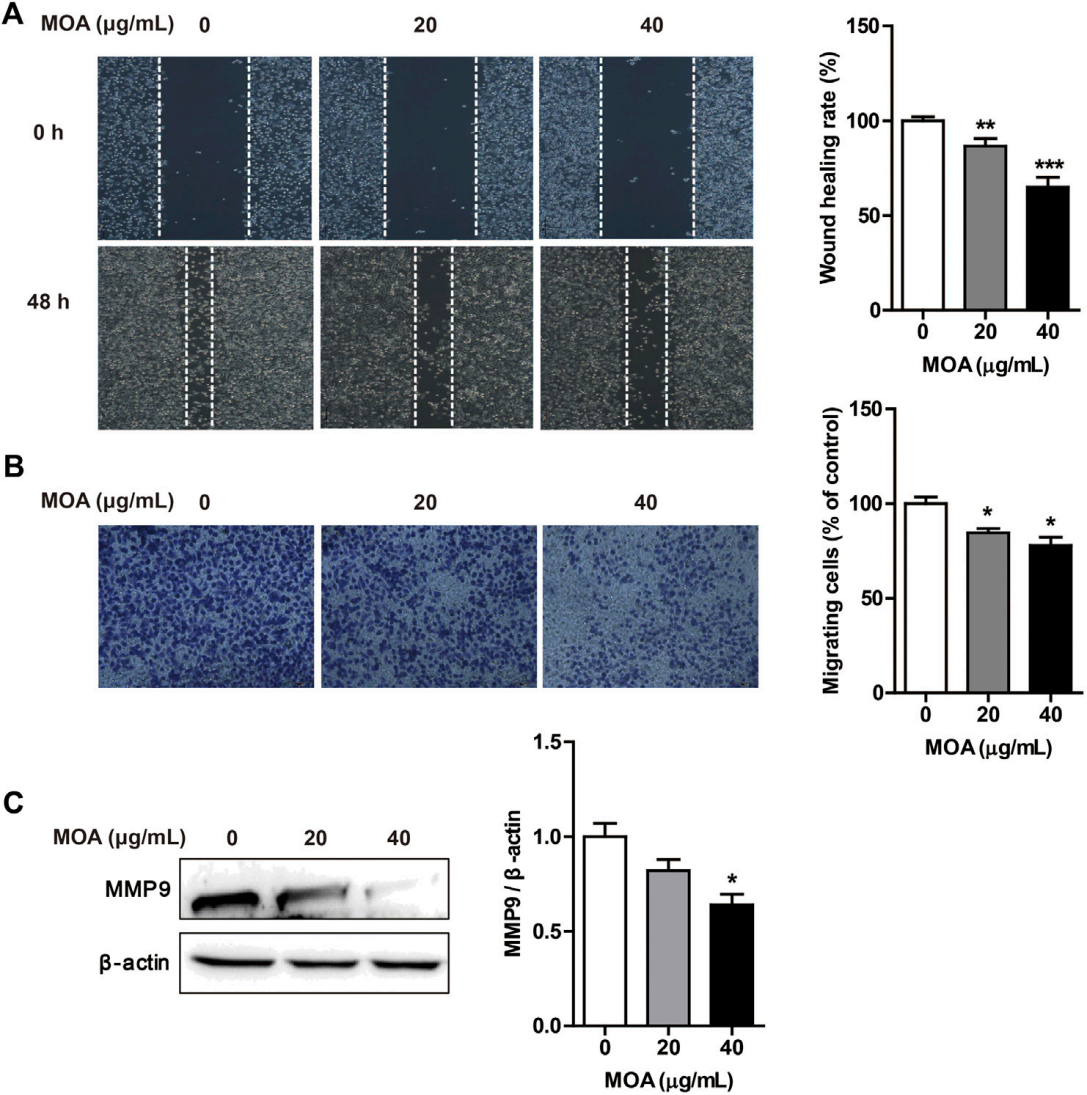
Treatment with MOA inhibited PC3 cell migration. PC3 cells were treated with MOA (0, 20, and 40 μg/ml) for 48 h, and cell migration was assessed using the wound healing assay **(A)** and transwell assay **(B)**. **(C)** The migration protein MMP9 was detected by western blotting, with β-actin as a loading control. Results are expressed as the mean ± SEM of three independent experiments. *p < 0.05, **p < 0.01, and ***p < 0.001 vs. 0 μg/ml.

*Cancer* cells typically show strong migration ability. We evaluated the effects of MOA on PC3 cell migration using the wound healing and transwell assays. The results showed that MOA significantly suppressed migration of the PC3 cells (Figure 3A). Furthermore, the transwell migration assay showed that MOA significantly weakened the migration ability of PC3 cells (Figure 3B). We then examined the expression levels of migration-related proteins using western blotting. The results showed that MOA (40 μg/ml) significantly decreased the expression levels of MMP9 (*p* < 0.05) in PC3 cells compared with those in control cells (Figure 3C). These results suggested that MOA inhibited PC3 cell migration.

### *M. oleifera* alkaloids Inhibited the Expression of Cyclooxygenase-2 and Reduced the Production of Prostaglandin E_2_ in PC3 Cells

**FIGURE 4:**
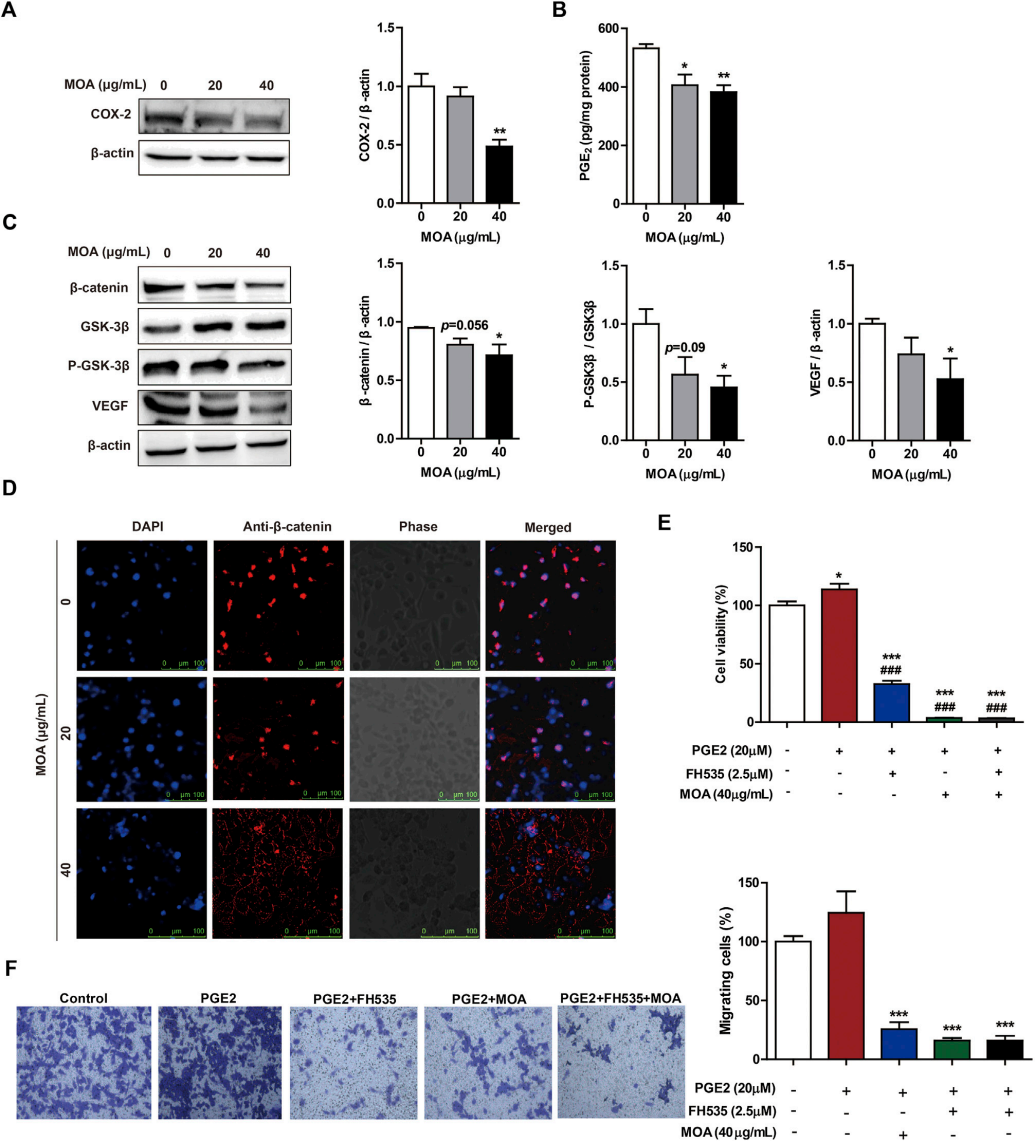
Treatment with MOA inhibited PC3 cell proliferation and migration via the COX-2 mediated Wnt/β-catenin signaling pathway. Western blotting was used to examine the protein levels of COX-2 **(A)**, β-catenin, GSK-3β, P-GSK-3β, and VEGF **(C)** in PC3 cells treated with MOA (0, 20, and 40 μg/ml) for 48 h. **(B)** The levels of PGE_2_ were measured in cell lysates using a PGE_2_ immunoassay kit. **(D)** Subcellular localization of β-catenin protein was determined using immunofluorescence. **(E,F)** Cell viability and cell migration were analyzed using the MTT and transwell assays. PC3 cells were treated with PGE_2_ (20 μM) and MOA (40 μg/ml), with or without FH535 (2.5 μM) for 48 h. The results are expressed as the mean ± SEM of three independent experiments. *p < 0.05, **p < 0.01, and ***p < 0.001 vs. 0 μg/ml, ^###^ *p* < 0.001 vs. PGE_2_ group.

To determine whether MOA inhibited PC3 cell proliferation and migration through modulation of COX-2 expression, we determined the levels of COX-2 in PC3 cells using western blotting. The results showed that MOA significantly decreased the expression of COX-2 compared to that in untreated control cells (Figure 4A). We then quantified PGE_2_, a major arachidonic acid metabolite produced by COX-2. The results showed that treatment with 20 and 40 μg/ml MOA reduced the levels of PGE_2_ in PC3 cells from 531.86 ± 14.60 pg/mg protein in control cells to 406.06 ± 36.42 pg/mg protein (*p* < 0.05) and 382.00 ± 24.01 pg/mg protein (*p* < 0.01), respectively (Figure 4B). These results showed that MOA suppressed proliferation and migration of PC3 cells by inhibiting the expression of COX-2, and reducing production of PGE_2_.

### Treatment With *M. oleifera* alkaloids Inhibited PC3 Cell Proliferation and Migration Via Cyclooxygenase-2 Mediated Wnt/β-Catenin Signaling Pathway

Studies have shown that activation of the β-catenin signaling pathway by PGE_2_ contributes to growth, invasion, and metastasis of cancer cells. To further explore the mechanisms underlying MOA-mediated inhibition of growth and migration of PC3 cells, the expression levels of β-catenin signaling pathway proteins were assessed using western blotting. Treatment with MOA decreased the expression of β-catenin, P-GSK-3β, and VEGF in a dose-dependent manner (Figure 4C). Furthermore, confocal fluorescence microscopy showed that the expression of β-catenin in the nucleus of MOA-treated PC3 cells was lower than that in untreated control cells (Figure 4D).

To determine the role of PGE_2_ and β-catenin in PC3 cell proliferation and migration, we treated cells with PGE_2_ (20 μM) and FH535 (2.5 μM) (an inhibitor of β-catenin) for 48 h, and measured cell proliferation and migration using the MTT and transwell assays. The results showed that PGE_2_ significantly enhanced proliferation (*p* < 0.05) and migration (*p* < 0.05) of PC3 cells, and FH535 significantly inhibited PGE_2_-mediated proliferation (*p* < 0.001) and migration (*p* < 0.001) of PC3 cells (Figures 4E,F). We then treated PC3 cells with PGE_2_ (20 μM) and MOA (40 μg/ml), with or without FH535 (2.5 μM), for 48 h. The results showed that MOA significantly inhibited PGE_2_-mediated proliferation (*p* < 0.001) and migration (*p* < 0.001) of PC3 cells, and the inhibitory effects of MOA and FH535 on PGE_2_-mediated proliferation and migration were not additive (Figures 4E,F). These results demonstrated that MOA inhibited PC3 cell growth and migration through the COX-2 mediated Wnt/β-catenin signaling pathway.

### *M. oleifera* alkaloids Inhibited PC3 Cell Growth Via the Cyclooxygenase-2-Mediated Wnt/β-Catenin Signaling Pathway *In Vivo*

**FIGURE 5:**
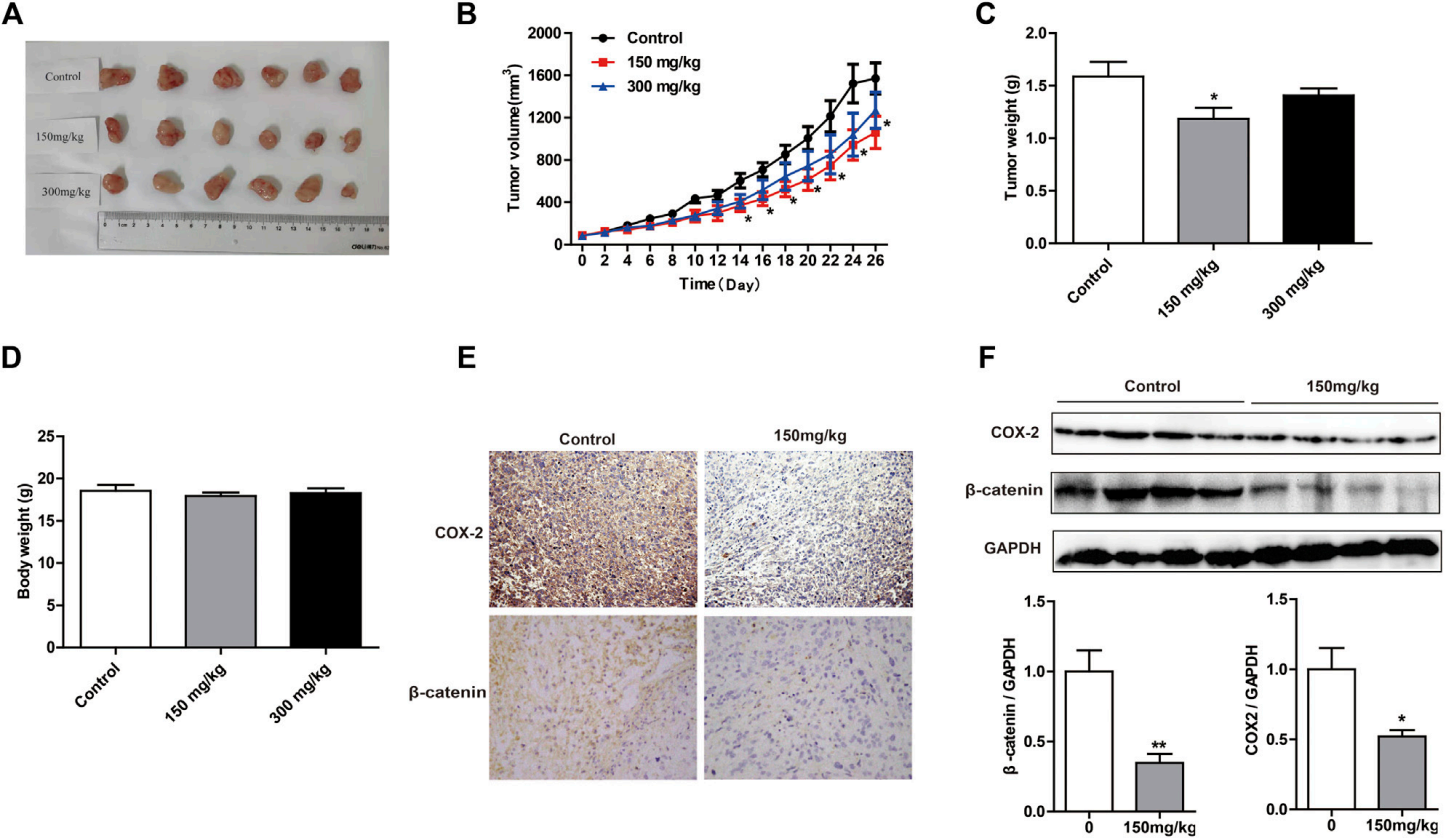
Treatment with MOA inhibited growth of subcutaneous xenograft tumors *in vivo*. **(A)** Photographs of tumors derived from nude mice in the control, 150 and 300 mg/kg MOA groups (*n* = 6 per group). Changes in tumor volumes **(B)**, tumor weights **(C)**, and body weights **(D)** from the three groups of nude mice. **(E)** Representative photographs of IHC staining of COX-2 and β-catenin in tumors from nude mice. **(F)** Western blotting analysis was used to determine the protein levels of COX-2 and β-catenin, with GAPDH as a loading control. Results are expressed as the mean ± SEM of three independent experiments. *p < 0.05 and **p < 0.01 vs. control.

To evaluate the inhibitory effects of MOA on tumor growth *in vivo*, we used a xenograft tumor model. As shown in Figures 5A,B, tumor volume was significantly decreased in response to MOA (150 mg/kg) treatment from day 14. Treatment with MOA (150 mg/kg) significantly decreased tumor weights from 1.59 ± 0.14 *g* in the control group to 1.16 ± 0.10 *g* (*p* < 0.05), which was a tumor inhibition rate of 25.16% (Figure 5C). Comparison of body weights indicated that treatment with MOA did not induce toxicity (Figure 5D). To determine whether MOA inhibited PC3 cell growth via COX-2-mediated Wnt/β-catenin signaling *in vivo*, we determined the protein levels of COX-2 and β-catenin in tumor tissues using immunohistochemistry and western blotting. The results showed that treatment with MOA significantly decreased the expression levels of COX-2 (*p* < 0.05) and β-catenin (*p* < 0.01) as compared to those in the control group (Figures 5E,F). These results suggested that MOA inhibited PC3 cell xenograft tumor growth via the COX-2-mediated Wnt/β-catenin signaling pathway.

## Discussion

*M. oleifera* is a traditional herbal medicine in India, and a large number of studies have reported that the extracts of various parts of *M. oleifera* (leaf, stem bark, pods, and seeds) inhibit growth of tumor cells. Water extracts of *M. oleifera* leaves have been shown to inhibit growth of lung cancer, liver cancer (Jung et al., 2015), oral cancer (Sreelatha and Padma, 2011), pancreatic cancer (Berkovich et al., 2013), and esophageal cancer cells (Tiloke et al., 2016). Ethanol extracts of *M. oleifera* leaves have been shown to inhibit growth of breast cancer (Diab et al., 2015), colon cancer (Al-Asmari et al., 2015), leukemia (Diab et al., 2015), and cervical cancer cells (Atawodi et al., 2010). Ethanol extracts of *M. oleifera* stem bark have been shown to inhibit growth of breast cancer cells (Al-Asmari et al., 2015). Water extracts of *M. oleifera* pods have been shown to inhibit growth of colon cancer cells (Budda et al., 2011). *M. oleifera* seed oil has been shown to inhibit growth of breast cancer cells (Diab et al., 2015). In addition, active compounds such as polyphenols and flavonoids in *M. oleifera* leaves and seeds have been shown to exert anti-tumor effects (Khor et al., 2018), and MOA have been hypothesized to be potential anti-cancer agents (Elsayed et al., 2015). In this study, we demonstrated that MOA inhibited growth and migration of prostate cancer PC3 cells *in vitro* and *in vivo*, and this effect resulted from inhibition of COX-2-mediated activation of the β-catenin signaling pathway.

Alkaloids are a class of organic nitrogen-containing compounds that exert analgesic, anti-spasmodic, anti-bacterial, anti-inflammatory, blood pressure-lowering, anti-asthmatic, and anti-tumor effects. The anti-tumor effects of alkaloids are potent, and these compounds exhibit low toxicity, which has led to increased interest in alkaloids as therapeutic agents. Researchers in China have screened more than 1000 natural medicinal plants for anti-tumor effects, and nearly 100 Chinese herbs have been shown to exert anti-tumor effects. Studies have shown that these anti-tumor effects may be largely due to the effects of alkaloids. Few studies have evaluated the activity of MOA. One study showed that the MOA α-l-rhamnosyl oligoglycoside isolated from *M. oleifera* leaves exerted cardioprotective effects (Panda et al., 2013; Cheraghi et al., 2017). MOA-trigonelline has been used to treat hyperglycemia, hyperlipidemia, insulin resistance, and diabetic auditory neuropathy (Oldoni et al., 2019); MOA-benzylamine decreased weight gain, fasting plasma glucose, and TC and increased glucose tolerance in insulin-resistant C57BL/6 mice (Iffiú-Soltész et al., 2010). However, the anti-tumor activity of MOA and their main active ingredients have not been characterized. In this study, we found that MOA inhibited growth and migration of PC3 cells. Although we performed LTQ-Orbitrap high resolution mass spectrometry analysis on MOA, some highly abundant compounds were not identified. Therefore, further systematic analysis of MOA is needed to identify other bioactive components.

Although many studies have proved that alkaloids have serious toxicity, there are also a large number of research have found that alkaloids have very little toxicity to normal cells. Noscapine is an alkaloid that inhibited the growth of breast cancer MCF-7 and MDA-MB-231 cells by inducing apoptosis, but is less toxic to normal breast cancer cell lines (MCF-10F); Papaverine and colchicine inhibited the growth of HT29, T47D, and HT1080 cell lines by inducing apoptosis, but they are not toxic to normal mouse embryonic fibroblast NIH3T3 cell lines. In addition, the alkaloids of evodiamine and rutaecarpine, which isolated from *Evodia rutaecarpa*, inhibited the proliferation of A375-S2, HeLa, MCF-7, THP-1, and L929 cells by inducing apoptosis, but these alkaloids did not impart any toxicity in normal human peripheral blood mononuclear cells (Mondal et al., 2019). In this study, we also found that MOA (20–80 μg/ml) did not impart any toxicity toward human normal prostate RWPE-1 cells. Interestingly, MOA at a dose of 150 mg/kg had a significant inhibitory effect on the tumor size and weight of PC3-inoculated mice, however, when the dose of MOA reaches 300 mg/kg, it had no significant effect on the tumor size and weight. We did notice that tumors in the sacrificed mice treated with high dosage of MOA started ulcerating. The results suggest that MOA may suppress the tumor growth of PC3 at low dose but exhibit no antitumor effect at high dose due to interruption of the inflammation balance of mice.

Studies have shown that alkaloids such as atrine (Huang et al., 2017), tetrandrine (Kou et al., 2016), mahanine (a plant-derived carbazole alkaloid) (Sinha et al., 2006), alpha-chaconine (a biologically active compound in potato extracts) (Reddivari et al., 2010), and berberine (Huang et al., 2015) inhibited invasion and induced apoptosis of prostate cancer cells. These studies indicated that alkaloids may be potential agents for treatment of prostate cancer. In this study, we found that MOA inhibited PC3 cell proliferation in a dose-dependent manner, as determined using the MTT and colony formation assays. Apoptosis is widely believed to be the major antiproliferative mechanism of anticancer drugs in many tumor cell types, and cell cycle is a determinant factor of cell growth and division. Therefore, in this study, we further analyzed the effects of MOA on cell apoptosis and cell cycle arrest of PC3 cells. We found that the percentage of apoptotic PC3 cells increased significantly, and cells were arrested in S phase, following treatment with MO. Western blot analysis showed that MOA significantly promoted Bax expression while suppresed Bcl-2 expression. Bcl-2 family played an essential role in cellular apoptotic process, in which Bcl-2 was with anti-apoptotic activity, whereas Bax was a pro-apoptotic member (Ding et al., 2011). Therefore, the results showed that MOA inhibited PC3 cell proliferation by inducing cell apoptosis. In addition, MOA treatment decreased the expression of cyclin E while enhanced the expression of p21. In the regulation of cell cycle G1/S phase, the network system of cyclins, cyclin-dependent kinases (CDKs), and cyclin-dependent kinases inhibitors (CDKIs) plays an important role in this process. CyclinE is a member of the cyclin protein family, and an important positive regulator involved in the G1/S conversion of the cell cycle. P21 was a CDKIs which bound to and inhibited the activity of cyclin-CDK complexes. In our study, the decreased expression of cyclinE and increased expression of p21 by MOA treatment indicated that cell cycle was arrest by MOA, which was responsible for the inhibitory effect of MOA on PC3 cells proliferation.

Cell migration plays an important role in many physiological and pathological processes, such as tissue repair and tumor cell metastasis. In this study, MOA significantly inhibited PC3 cell migration by using the wound healing and transwell assays. Matrix metalloproteinases (MMPs) promote tumor cell invasion and metastasis by breaking down the basement membrane and extracellular matrix. MMP2 and MMP9 are members of the MMPs family, and they have the function of decomposing the collagen of basement membrane. It is known that the malignant degree of different types of tumors is positively correlated with the excessive expression of MMP2 and MMP9. In this study, MOA significantly decreased the expression of MMP9. The results suggest that MOA suppressed PC3 cell migration and inhibited the expression of MMP9.

Onset and development of tumors are closely related to increased expression of COX-2 (Hashemi Goradel et al., 2019). Many studies have shown that COX-2 is overexpressed during progression of prostate cancer (Gupta et al., 2000; Dandekar and Lokeshwar, 2004; Richardsen et al., 2010). In addition, PGE_2_, an arachidonic acid metabolite produced by COX-2, is a mitogen and contributes to development of prostate cancer (Madrigal-Martinez et al., 2019). Therefore, targeting COX-2 is a promising strategy for treatment of prostate cancer. We evaluated the inhibitory effect of MOA on COX-2 in prostate cancer PC3 cells *in vitro* and *in vivo*. The results showed that MOA significantly inhibited COX-2 protein expression in PC3 cells, and significantly inhibited PGE_2_ secretion. These results indicated that MOA inhibited PC3 cell growth and migration through inhibition of COX-2, and subsequent inhibition of production of PGE_2_.

The Wnt signaling pathway plays an important role in cell proliferation, differentiation, migration, apoptosis, and tumor development. Disruption of the classical Wnt/β-catenin pathway is one of the most common abnormalities associated with human malignancies (Nusse and Clevers, 2017). Abnormal activation of the Wnt/β-catenin signaling has been shown in a number of malignancies including colon cancer, prostate cancer, ovarian cancer, and breast cancer (Mohammed et al., 2016). Activation of the classical Wnt signaling pathway results in stabilization of β-catenin in the cytoplasm, and subsequent translocation to the nucleus (Shi et al., 2015). In the absence of Wnt ligands, cytoplasmic β-catenin is degraded by a complex comprised of the scaffold protein Axin, adenomatous polyposis *coli* (APC), GSK3β, and casein kinase 1 (CK1). This prevents β-catenin from entering the nucleus, which prevents transcription of downstream target genes (Dihlmann and von Knebel Doeberitz, 2005). In the presence of Wnt ligands, Wnt binds to receptors on the cell membrane and recruits Axin to the cell membrane, which results in dissociation of the degradation complex. This dissociation allows free β-catenin to accumulate in the cytoplasm and to enter the nucleus. In the nucleus, β-catenin binds to the transcription factor T cell factor/lymphoid enhancer factor (TCF/LEF) to promote transcription of downstream target genes (Xu et al., 2016). A previous study found that the Wnt/β-catenin pathway played a key role in the pathogenesis of prostate cancer through regulation of angiogenesis, drug resistance, cell proliferation, and apoptosis (Pashirzad et al., 2019). Another study showed that β-catenin was not expressed, or was expressed at low levels, in normal prostate tissue, but was highly expressed in prostate cancer tissues (Chen et al., 2004). In our study, we showed that MOA inhibited the expression of β-catenin *in vitro* and *in vivo*. In addition, MOA significantly inhibited phosphorylation of GSK3β in a dose-dependent manner, which indicated that MOA inhibited the Wnt/β-catenin signaling pathway.

Numerous studies have shown a link between COX-2/PGE_2_ and Wnt/β-catenin signaling in development of many cancers, including lung cancer (Singh and Katiyar, 2013), skin cancer (Prasad and Katiyar, 2014), breast cancer (Tu et al., 2014), colorectal cancer (Castellone et al., 2005), and neuroblastoma (Jansen et al., 2015). In addition, a number of studies have shown that the COX-2/PGE_2_/β-catenin signaling pathway may be a potential target to inhibit cancer cell growth and migration. For example, tanshinone IIA has been shown to downregulate VEGF levels by inhibiting the expression of COX-2 and Wnt/β-catenin signaling, which resulted in inhibition of colon cancer cell growth (Ma et al., 2018). Total flavonoids from Radix Tetrastigmae have been shown to inhibit Hep-G2 cell proliferation through inhibition of the COX-2/Wnt/β-catenin signaling pathway (Qinglin et al., 2017). Furthermore, meloxicam has been shown to inhibit proliferation and migration of hepatoma cells by targeting COX-2/PGE_2_ to regulate activation of the β-catenin signaling pathway (Li et al., 2016). In addition, magnolol has been shown to inhibit migration of non-small cell lung cancer cells by targeting PGE_2_ to regulate activation of the β-catenin signaling pathway (Singh and Katiyar, 2013). In our study, MOA inhibited PC3 cell proliferation and migration by inhibiting the COX-2/PGE_2_/β-catenin signaling pathway.

## Data Availability

All datasets generated for this study are included in the article/supplementary material.
