# Corrigendum: *Moringa oleifera* Alkaloids Inhibited PC3 Cells Growth and Migration Through the COX-2 Mediated Wnt/β-Catenin Signaling Pathway

**DOI:** 10.3389/fphar.2021.760933

**Published:** 2021-09-16

**Authors:** Jing Xie, Feng-xian Luo, Chong-ying Shi, Wei-wei Jiang, Ying-yan Qian, Ming-rong Yang, Shuang Song, Tian-yi Dai, Lei Peng, Xiao-yu Gao, Liang Tao, Yang Tian, Jun Sheng

**Affiliations:** ^1^ College of Food Science and Technology, Yunnan Agricultural University, Kunming, China; ^2^ Engineering Research Center of Development and Utilization of Food and Drug Homologous Resources, Ministry of Education, Yunnan Agricultural University, Kunming, China; ^3^ National Research and Development Professional Center for Moringa Processing Technology, Yunnan Agricultural University, Kunming, China; ^4^ College of Science, Yunnan Agricultural University, Kunming, China; ^5^ Yunnan Province Engineering Research Center of Functional Food of Homologous of Drug and Food, Yunnan Agricultural University, Kunming, China; ^6^ Key Laboratory of Pu-er Tea Science, Ministry of Education, Yunnan Agricultural University, Kunming, China

**Keywords:** *Moringa oleifera* alkaloids, prostate cancer, PC3 cells, cell growth and migration, COX-2-wnt/β-catenin signaling pathway

In the original article, there was a mistake in Figure 1C as published. Some images in Figure 1C were mistakenly duplicated. The corrected Figure 1 appears below.

**FIGURE 1:**
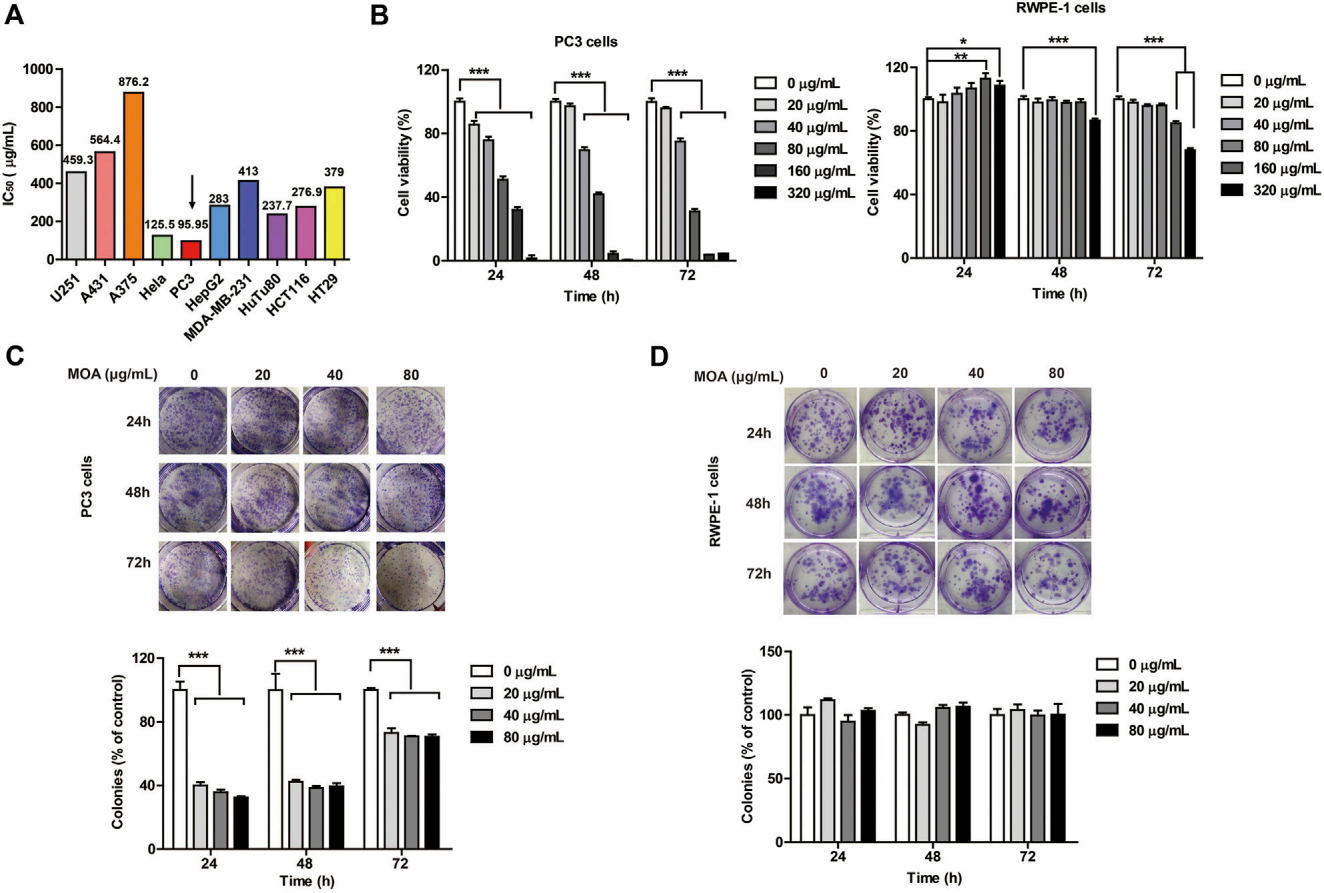
The effect of MOA on the proliferation of PC3 and RWPE-1 cells. **(A)** The half maximal inhibitory concentration (IC_50_) of ten tumor cell lines following treatment with MOA (0–320 μg/ml) for 48 h. **(B)** Cell viability of PC3 and RWPE-1 cells following MOA (0–320 μg/ml) treatment for 24, 48, and 72 h. **(C,D)** Analysis of the colony formation ability of PC3 cells following treatment with MOA (0, 20, 40, and 80 μg/ml) for 24, 48, and 72 h. The cellular colony formation rates are expressed as fold changes. Results are expressed as the mean ± SEM of three independent experiments. ***p < 0.001 vs. 0 μg/ml.

The authors apologize for this error and state that this does not change the scientific conclusions of the article in any way. The original article has been updated.

